# A Smart Sensing Architecture for Domestic Monitoring: Methodological Approach and Experimental Validation

**DOI:** 10.3390/s18072310

**Published:** 2018-07-17

**Authors:** Andrea Monteriù, Mario Rosario Prist, Emanuele Frontoni, Sauro Longhi, Filippo Pietroni, Sara Casaccia, Lorenzo Scalise, Annalisa Cenci, Luca Romeo, Riccardo Berta, Loreto Pescosolido, Gianni Orlandi, Gian Marco Revel

**Affiliations:** 1 Department of Information Engineering, Università Politecnica delle Marche, 60131 Ancona, Italy; m.prist@univpm.it (M.R.P.); e.frontoni@univpm.it (E.F.); s.longhi@univpm.it (S.L.); a.cenci@univpm.it (A.C.); l.romeo@univpm.it (L.R.); 2 Department of Industrial Engineering and Mathematical Sciences, Università Politecnica delle Marche, 60131 Ancona, Italy; f.pietroni@univpm.it (F.P.); s.casaccia@univpm.it (S.C.); l.scalise@univpm.it (L.S.); gm.revel@univpm.it (G.M.R.); 3 Electrical, Electronics and Telecommunication Engineering and Naval Architecture Department, Università degli Studi di Genova, 16145 Genoa, Italy; riccardo.berta@unige.it; 4 Italian National Research Council, Institute for Informatics and Telematics (CNR-IIT), 56124 Pisa, Italy; loreto.pescosolido@iit.cnr.it; 5 Department of Information Engineering, Electronics e Telecommunications, University of Rome La Sapienza, 00184 Rome, Italy; gianni.orlandi@uniroma1.it

**Keywords:** smart homes, smart home technologies, aging, activity of daily living

## Abstract

Smart homes play a strategic role for improving life quality of people, enabling to monitor people at home with numerous intelligent devices. Sensors can be installed to provide a continuous assistance without limiting the resident’s daily routine, giving her/him greater comfort, well-being and safety. This paper is based on the development of domestic technological solutions to improve the life quality of citizens and monitor the users and the domestic environment, based on features extracted from the collected data. The proposed smart sensing architecture is based on an integrated sensor network to monitor the user and the environment to derive information about the user’s behavior and her/his health status. The proposed platform includes biomedical, wearable, and unobtrusive sensors for monitoring user’s physiological parameters and home automation sensors to obtain information about her/his environment. The sensor network stores the heterogeneous data both locally and remotely in Cloud, where machine learning algorithms and data mining strategies are used for user behavior identification, classification of user health conditions, classification of the smart home profile, and data analytics to implement services for the community. The proposed solution has been experimentally tested in a pilot study based on the development of both sensors and services for elderly users at home.

## 1. Introduction

Life expectancy has increased significantly in the last years thanks to the rapid growth in medical science. The number of people aged 65 and older is increasing [1]. Consequently, the percentage of elderly people that prefer to stay in their homes and communities is growing: this phenomenon is called “Aging in Place” [2]. In this context, smart home technologies could significantly support people to have a better life quality, to live independently and to stay in contact with family and caregivers [3]. Thanks to their software and hardware components, smart homes allow collectin data and, thus, to monitor the health status, the behavior and the life quality of elderly users, avoiding risky situations and putting the users in contact with their family, caregivers and medical staff [4]. Thus, modern sensor-embedded houses, or smart houses, can not only assist elderly people but also help to resolve the social isolation they could face [5,6]. In this scenario, smart home technologies hold a great promise for the future of healthcare and well-being of older adults, people with disabilities, and the general population overall [7].

Smart homes have been extensively researched in the last years. The “Smart Rooms” implemented by the MIT Media Lab [8] could be defined as the first approach to this topic. Thereafter, several papers have been published on this topic with a wide range of prospective applications. A review of the state of the art of smart homes is presented in [9,10,11], while an international selection of leading smart home projects, as well as the associated technologies of wearable/implantable monitoring systems and assistive robotics are presented in [5]. A review of sensor technology used in smart homes with a focus on direct environment sensing and infrastructure mediated sensing is provided in [12]. A review of the potential of smart homes to support independent living is provided in [13], identifying some of the health needs of elderly people who could live at home if provided with adequate support, the range and type of technologies that could be employed to this objective, and suitable metrics to be used to measure the effectiveness of these technologies. Many research projects have been delivered on smart homes with the objective to provide an ecosystem of medical and home automation sensors, computers or smartphone, wireless networks [14] and software services for healthcare monitoring and control [5]. Many of them can be grouped into distinct categories, such as projects for facilitating the connection between the elderly and their informal caregiver, projects for monitoring health parameters and generate alerts messages in relation to a specific programmable situation, or projects that realize the smart home monitoring with the focus to learn and to predict the habits of its inhabitants in order to manage elderly safety and improve life quality. The use of a new architecture and technologies for intelligent environments, which includes smart user interface, dynamic configuration of the smart sensors, wireless control, tracking of the elderly people and flexible user services interaction, was proposed by Brumitt et al. in the *EasyLiving* project [15] at Microsoft Research. A multi-disciplinary research project, namely the *CASAS Smart Home* project, has been presented by the Washington State University. The objective of the project was to improve the safety, the life quality, the comfort and the state of the smart home residents using intelligent software agents and sensors and environment controllers [16,17]. In [18], Cook et al. proposed an intelligent and versatile home environment, namely *MavHome* project, which acquires data, and learns and predicts the residents habits to maximize their comfort and wellness, while reducing operating cost. The *DOREMI* project [19] is oriented to reduce the cognitive decline, malnutrition, and sedentariness of elderly people, and to increase the social interaction with the use of cognitive virtual games and virtual companion. The use of software platform and smart object permits to stimulate and to unobtrusively monitor the elderly daily life activities. In addition, different AAL platforms and solutions exist and have been studied in different European projects, see for instance [20,21,22,23,24]. Although the described smart home projects apply very interesting concepts in real scenarios, they often do not integrate all technological aspects such as biomedical sensors, wireless sensor network, home automation devices, behavioral analysis, Cloud infrastructure, custom Cloud services and flexibility, interoperability and extensibility of the acquisition software platform.

This paper is focused on the development of domestic technological solutions to improve the life quality of citizens and monitor the users and the environment where they spend most of their time, i.e., their house, based on features extracted from the collected data. The innovative system described in this paper is based on an integrated sensor network to monitor the user and the environment to derive information about the user’s behavior, her/his health status, her/his social condition, etc. The proposed platform includes biomedical, wearable, and unobtrusive sensors for monitoring user’s physiological parameters and home automation sensors to obtain information about her/his environment, e.g., energy consumptions, light status, user movement, etc. The sensor network stores the heterogeneous data both locally and remotely in Cloud, where machine learning algorithms and data mining strategies are used for user-health behavior identification, classification of user health conditions, classification of the smart home profile, and data analytics to implement services for the community. One of the aspects of our proposed architecture with respect to the existing ones is the user interaction with the architecture, namely the way the user can interact with the system by using, as a user interface, an Android app that can be run on most commercial smartphones/tablets to perform biomedical measurements from any of the devices. More specifically, the app layout depends on the specific logged-in user in a dynamic fashion. Each user is presented with the subset of devices, among those present in the house, which she/he has the permission to use. The app serves to initiate the measurement process from any of such devices. The proposed solution has been experimentally tested in a pilot study carried out in a small city of Veneto Region, Italy, within the context of the Health@Home project [25]. The pilot involved the development of both sensors and services for elderly users at home. Thus, the ICT technologies, characteristics of a smart home, are used to collect data and information, with the aim to provide ad-hoc services (e.g., care and social services) for elderly users and to improve their quality of life. It is worth pointing out that the idea of bringing integrated monitoring services to citizens’ homes, is not confined to the vertical e-Health domain. In fact, the concept of “Smart Home” is well integrated in horizontal domains such as “Smart Buildings” and “Smart Cities” [7,26,27]. The Health@Home project platform aims at the overall development of an e-market place where services are offered, which make use of citizen’s data collected in their home environments. The ultimate goal of the project is to generate service models that are economically sustainable by coupling social/sanitary support (e.g., catering, social assistance, etc.) with house-related interventions (e.g., maintenance of appliances) exploiting a unique ICT platform. This is the reason for the sensor network architecture proposed in this paper, here specifically exploited for advanced domestic monitoring as a part of the whole system.

The paper is organized as follows. The hardware and software of the proposed smart sensing architecture are described in Section 2. The pilot case is detailed in Section 3, while an extensive analysis of the results of the pilot study is presented in Section 4. Some remarks and future works conclude this paper in Section 5.

## 2. The Proposed Smart Sensing Architecture

**Figure 1:**
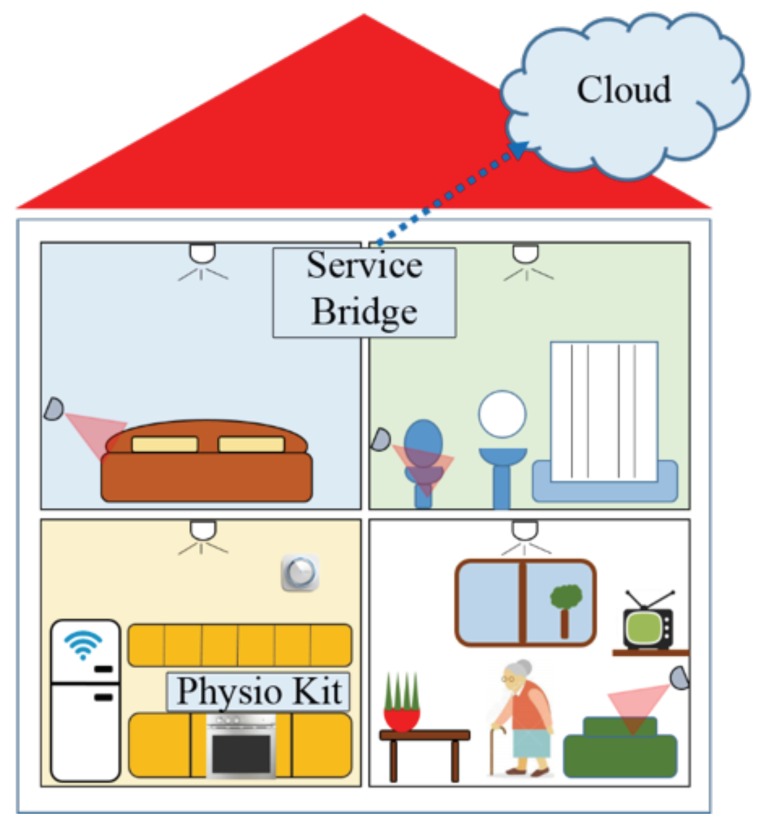
The proposed Smart Home architecture.

The hardware and software components of the smart home designed and developed for our pilot study, are presented in this section. The proposed smart sensing architecture is sketched out in Figure 1. The hardware is an integrated sensor network composed by: devices to acquire and monitor the physiological parameters of the user (i.e., the *Physio Kit*); sensors to acquire and monitor the user domestic environment (i.e., the *Home Automation Kit*); a gateway and local storage device, which is here called *Service Bridge*, to collect the data and signals; and a smart device (i.e., a tablet), with a simplified and accessible user interface to interact with the user for physiological data acquisitions and for controlling the home automation. Both physiological and environmental data collected by all sensors are sent to the Cloud to be stored and processed, as depicted in Figure 1.

In the following subsections, hardware and software of the proposed domestic sensing architecture are described in detail.

### 2.1. Smart Home Architecture: Hardware

#### 2.1.1. Sensors for Physiological Monitoring

**Figure 2:**
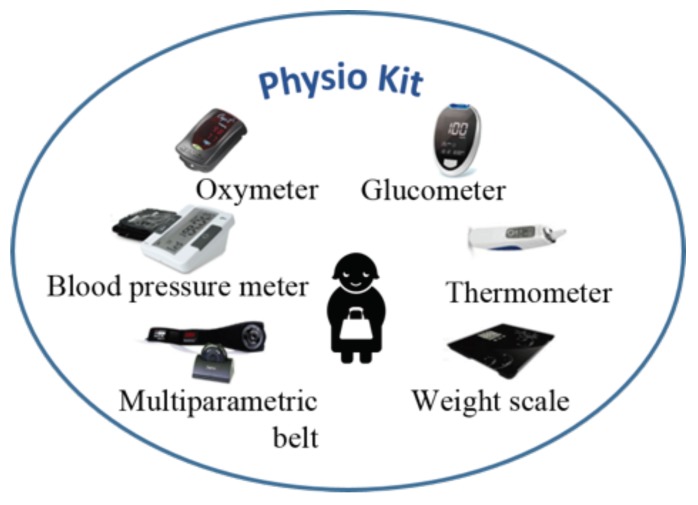
*Physio Kit* for the measurement of user’s health status.

The health status and well-being of a person is complex task. For this work, several fundamental quantities to be monitored have been identified to assess the health condition of a user. The selected physiological parameters include: Electrocardiogram signal (ECG), Heart Rate (HR), Breathing waveform, Breathing Rate (BR), Oxygen Saturation, Blood Pressure (BP), Body weight, Body Temperature, and Glycemia. The combination of these parameters can provide a general assessment of the health status of the user. To develop a technological system to help the user in doing the measurement at home without any assistance, unobtrusive and wearable biomedical devices have been chosen for the measurement setup, and a kit to measure the selected physiological parameters has been provided to each user. Wearable and non-invasive health sensors are integrated in the same kit, hereinafter called *Physio Kit* (see Figure 2).

The developed *Physio Kit* is characterized by devices satisfying several requirements. First, the sensors are easy to use for elderly people. The choice has fallen on devices that are familiar to the user (e.g., devices that are typically used with medical staff at hospitals, care centers, pharmacies, etc.). Moreover, all the devices are characterized by an adequate measurement accuracy, a wireless communication protocol (i.e., bluetooth) to avoid the need for the user to handle cables and plugs, and a low cost. Table 1 reports the description of the selected biomedical devices.

#### 2.1.2. Sensors for Environmental Monitoring

The developed *Home Kit* is the component of the home sensor network that allows monitoring the house and the environment. Aspects that have been identified to be of potential interest, e.g., to perform a comparative analysis with the data extracted by the biomedical sensors, are the behavior of the user inside the apartment (i.e., their sojourn times in each room of the apartment), the electrical consumption, air temperature and light status. The following devices from Bticino have been selected to monitor the house:

- **Thermostat** (LN4691) is a device for the temperature monitoring and control inside the apartment, equipped with a temperature probe.
- **Green switch**—**IR and ultrasound** (L4658N)—is a presence control system (Passive InfraRed-PIR). All smart homes are equipped with at least two PIR for each apartment. The authors are developing a tool to define the activities (behavior) of the older person inside the apartment using the ON/OFF signals coming from the PIR. The PIR are usually installed near the door of the bedroom and the bathroom and in some apartments in the kitchen, for others near the main door or in the living room based on the apartment plan.
- **Electrical consumption meters** (F520) are installed in the TV, the washing machine and the oven.
- **Light status** (F411/4) are used to control the ON/OFF of the lights to define, together with the PIR analysis, the behavior of the older user.

#### 2.1.3. Additional Smart Objects

To improve the overall information about the behavior of the users within their homes, a smart fridge from Whirlpool and a smart device for air quality control, called **SNAP**, from Elica have been integrated in the sensor network and installed in some of the apartments of the pilot case. In detail, the **Smart Fridge** (BSNF 8999 PB) is a two-compartment fridge with Wi-Fi technology to extract some information, e.g., open/close doors (fridge and refrigerator), fridge temperature and freezer temperatures (operative ranges: 2 to 8 ${}^{{}^{\circ}}$C, −16 to −24 ${}^{{}^{\circ}}$C, respectively). The SNAP is a device to control and manage the air quality (estimated through TVOC embedded sensor) and measure the temperature (−40 to 125 ${}^{{}^{\circ}}$C) and humidity of the environment (0% to 100%). The SNAP are installed in the kitchen, usually in the wall above the cooktop.

### 2.2. Smart Home Architecture: Software

**Figure 3:**
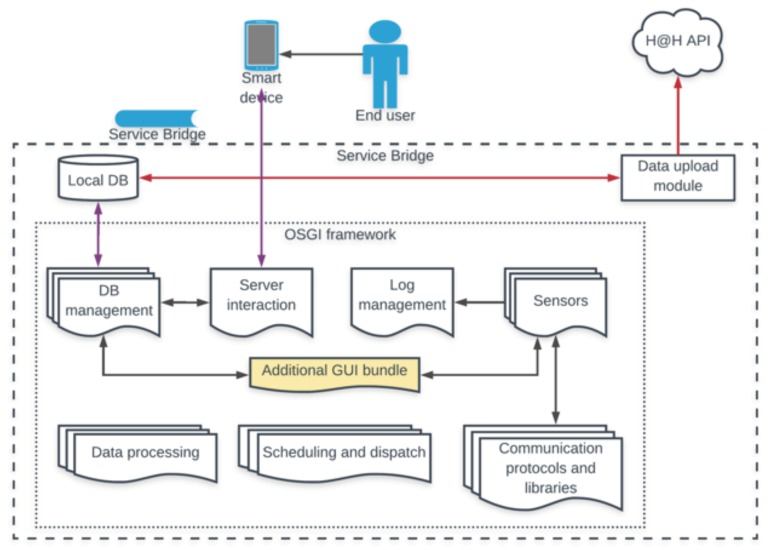
Concept of the Smart Home architecture developed and tested in the pilot case.

The concept of the software architecture adopted in this pilot study is resumed in Figure 3. The main development tool for the *Service Bridge* is based on the Open Service Gateway Initiative (OSGi) framework, with a set of bundles for managing all aspects of data acquisition and manipulation.

The non-profit OSGi was born in 1999 by union of IBM, Oracle, Philips, Sun, etc., with the aim to standardize the approach of modular applications development in the field of home and industrial automation [28]. The OSGi framework has the goal to implement a clear specification for building modular applications based on components and introduce a programming Service Oriented Architecture (SOA), allowing a separation much more rigorous than the other native paradigms, between the interface and its implementation. From the programming point of view, OSGi is a modular system for Java language, which defines the modality to create modules and how to interact with each other, at runtime. The OSGi architecture is composed by OSGi Framework and a set of bundles.

**Figure 4:**
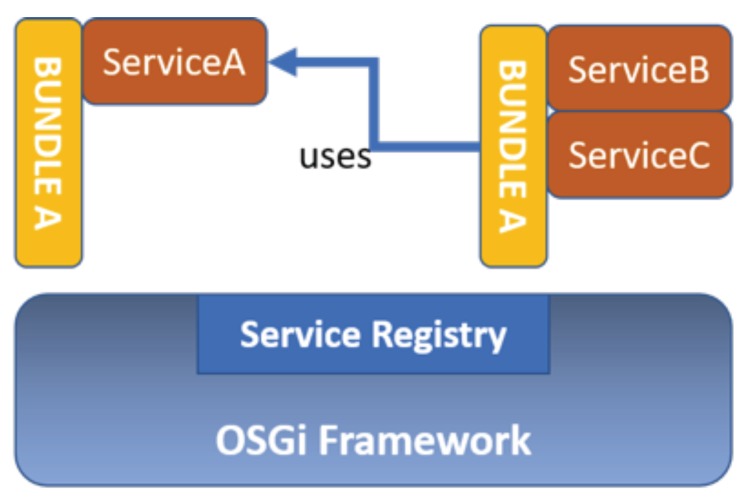
OSGi Framework with Bundles and Services.

OSGi implements a centralized service-oriented architecture with loosely coupled services (see Figure 4). The basic functionality is provided by the OSGi Framework to execute OSGi functions and the environment to download and run bundles. In addition, OSGi is a dynamic modular system where the modules can be installed, updated and uninstalled without affecting the entire application. OSGi is focused on providing developers with software tools aimed at endowing strong interoperability and modularity capabilities into the devised software. This was a major requirement for the selection of the framework to use in the development of different modules of the proposed software architecture, given also to the heterogeneity of the devices’ manufacturers. In addition, the *Service Bridge* itself exposes a server (exclusively accessible on the private Wi-Fi network of the apartment), which communicates with an Android App installed on a dedicated smart device, namely a tablet, and allows the end-user to perform the biomedical measurements, while interacting with the developed Graphical User Interface (GUI). The same Android App comprises an ad-hoc module, which allows the acquisition of all quantities and data from the sensor equipment, described before, sending them to the *Service Bridge*. Finally, data storage is performed both locally (i.e., with a local database in the *Service Bridge*) and on Cloud, using a dedicated data upload module. All these aspects are described in detail in the following subsections. This architecture has been continuously developed within the project and improved with respect to what described by authors in previous works [29,30].

#### 2.2.1. Back-End OSGI Framework

When implementing the proposed software architecture of the *Service Bridge*, the considered principal aspects have been modularity and extendibility, together with the possibility to integrate new sensors in a “plug and play” mode, only developing a new set of bundles without modifying the software architecture. In detail, the attention has been focused on the sub-modules related to the interface with the physical devices, to the local data storage, to data pre-processing, and to data exchange. As previously stated, to define a clear, simple and dynamic deployment format which enables run-time extendibility and allows very loose coupling between components, an OSGi Framework has been used. Thanks to this choice, this paper proposes an active approach to maintain the *Service Bridge* as transparently as possible, which means that user interactions are kept to the minimum required. The proposed approach provides a supervisor-bundle and a collection of bundles, which are related to sensors, devices, home automation system, database management, data analysis and external communication mechanisms. The supervisor-bundle represents the active extensible core to integrate sensors and user interface into the software platform. This core is also used for the management of security events related to sensors.

**Figure 5:**
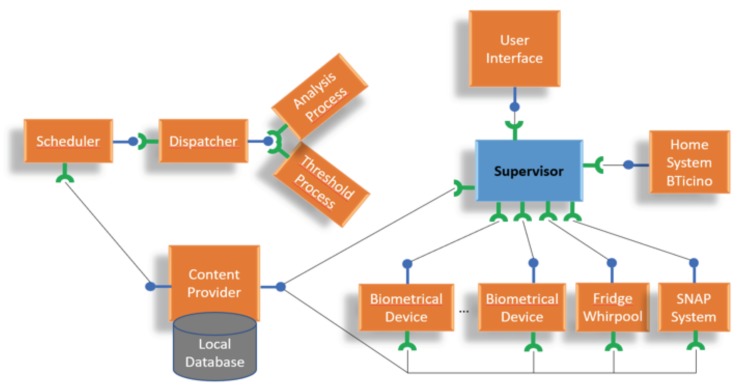
The developed bundles architecture.

Figure 5 shows the developed bundles architecture, where sensors and devices are involved in the monitoring of security-related events. In the proposed scenario, several combinations of sensors and devices are possible. A sensor can be active and directly sent data to supervisor, or it can be passive, requiring the user interaction to start the acquisition. The local database, which is used to save data coming from biometric devices and home automation system, before transmission to the Cloud, consists both of static and dynamic tables. While static tables include the collection of every device involved in the system, the dynamic ones contain data and statistics. Information stored into static tables include device ID, credential accesses, and statistical indicators. They are updated when two types of events are called: a new Cloud setting, or a new device added to the OSGi Framework (in this case, a new bundle is installed in the software). On the other hand, dynamic tables are updated in runtime. Therefore, while all raw data are available for the Cloud repository, statistical information is firstly elaborated by a local machine and, then, sent to Cloud Computing for further elaborations and analysis. Static tables also include information about the processing time for the statistical analysis. An asynchronous scheduler, integrated in a dedicated bundle, has been used to implement this functionality. This scheduler can additionally schedule commands to run after a given delay and to periodically execute biometric data processing, which increases computational efficiency. The number of tasks is directly related to the number of different devices installed into the *Service Bridge*, and to the number of indicators to be monitored. The proposed bundles architecture for the asynchronous scheduler (see Figure 5) consists of three levels: Scheduler, Dispatcher, and Threshold. The Scheduler module runs a thread when each event is verified. The Dispatcher module controls if the current event should be associated to a specific indicator and it notifies to all subscribed services to run the related data processing. The Threshold module executes the elaboration process. The implementation of each software-module has been compliant with the OSGi open standard [31,32].

#### 2.2.2. Front-End User Interface

In the pilot case experimentation, users have been equipped with an Android device, namely a tablet, and a simple user interface, developed ad-hoc. In this way, they can perform measurements from the *Physio Kit* (e.g., blood pressure) and then trigger the *Service Bridge* to collect the acquired data and to store them in the Cloud (see Section 2.3). The App, referred to as *Health App*, communicates directly with the *Service Bridge*, through a Server and several REpresentational State Transfer (REST) pages. The *Health App* has a user interface which is minimal and very accessible and simple to use.

**Figure 6:**
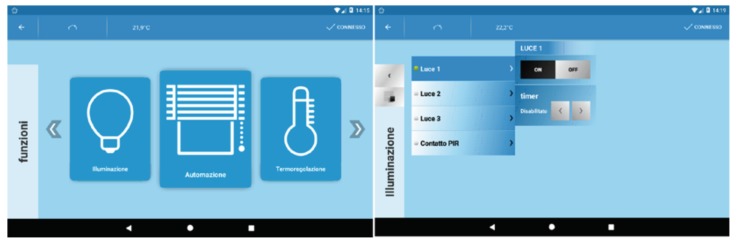
Snapshots of the *Home Automation App* for monitoring and control the environment.

When the *Health App* starts, the first section is the authentication. Once the user has provided the correct credentials (i.e., username and password which are stored in a local database, protected with OS-level encryption), the home page is shown. On the home page, a first binary option is presented to the user, in the form of two buttons: it is possible to either access a further page of the *Health App*, which is devoted to the control of the biomedical devices (i.e., it is used to perform measurements) or to access the domestic environment controls (e.g., to monitor the state of light, to turn on/off, etc.). If the first option is selected, a list of all the sensors of the *Physio Kit* installed in the apartment is shown to the user. It is then possible to activate the measurement process of every single biomedical device, by clicking the relative button and by following the instructions on the screen. For some devices, those who need to be activated manually by the user, the App just instructs the *Service Bridge* to wait for an incoming measurement from the user that is logged in. For other devices, which can be triggered remotely, the App instructs the *Service Bridge* to send the trigger to the device, in order to start the measurement. In our pilot experiment, users were trained to use this application, by means of documentation material provided by the experimenter and through in site demonstrations and tutorials. In summary, the *Health App* communicates with the *Service Bridge*, which is then demanded to all the following tasks, as described before (data acquisition, data storage to both local DB and Cloud, etc.). The user interface of the *Health App* has been kept as simple as possible, also on the basis of the feedback received by the volunteers that have been involved by us prior to the release of the App. Particularly, it is not possible, from the App, to see the measurements, as it is intended to just provide a means for users to let the measured values be stored in the local DB. The measurements can only be seen through a dedicated web service, which acts at the Cloud level (see Section 2.3). If, on the home page of the *Health App*, the user selects the environmental control, another App is started, hereafter called *Home Automation App*. The *Home Automation App* is able to automatically collect data (e.g., PIR triggers, lights on/off, and room temperature), without any additional required interaction. However, if wanted, the user may access to the information about the *Home Automation Kit* with the *Home Automation App* and, eventually, control the environment itself (e.g., turning on a light, as shown in Figure 6).

Note that the main reason for the development of the *Health App* has been the necessity to trigger the acquisition of the sensors. This is because of the non-feasibility, for the *Service Bridge* (i.e., for OSGI and the adopted architecture), to deal with the identification of collected devices (only one communication is allowed at each time). However, from the first conducted demos, some users found difficult to interact with a technology that they are not aware of. For example, the authentication part, developed to be in agreement with security standards, is not easily completed by all users. A second development stage has been necessary to provide a suitable solution for these persons. Thus, an Android box has been installed in some apartments. This device hosts both the home automation service (part of the previously described *Home Automation App*), to collect the environmental measurements, together with a dedicated scanning service. This service directly communicates with the *Service Bridge* (i.e., the server), scans both Bluetooth and BLE devices connected at the moment, and sends a command to start the acquisition (refer to Section 2.4 for the different user interaction flows). In this way, the acquisition can be conducted by the user, without the need to interact with the *Health App*.

### 2.3. Smart Home Architecture: Data Storage and Cloud Interaction

For the pilot study presented in this work, the previously described Smart Home architecture was used in combination with a Cloud-based architecture (also developed within the context of the Health@Home project [25]). The detailed description of the Cloud-based architecture is outside the scope of this work, which is focused on the Smart Home architecture. Here, only a sketch of its fundamental elements, with a focus on data storage and data access, is provided.

Data are organized around the abstract concepts of *Users*, *Devices*, *Features*, *Raw Measurements*, *Indicators*, and *Indicator values*:

- *User*: An end-user of the health monitoring services offered through the platform.
- *Device*: A device (environmental sensors, physiological parameters monitoring gadget, home automation appliances, etc.) which provides measurements about a user and stores the values on the Cloud.
- *Feature*: A physical dimension that can be measured by a device (e.g., the heart rate, the environment temperature, etc.).
- *Raw measurement*: The specific instance of a feature measurement, acquired by a device for a specific user, stored in the local database and on the Cloud, and accessible by any service (to which the user has subscribed) to implement its functions.
- *Indicators*: The definition of a particular function of a set of raw measurements (min, max, average, etc.) computed for one user over a predefined time window, with the goal to extract particular trends in the evolution of a physiological quantity value.
- *Indicator values*: The actual results of computations performed on a group of raw measurements, according to the definition of an “indicator”.

Two different levels of storage are present to save data coming from the different devices: the Smart Home and the Cloud level. The database implementation at both levels (SQL-based in the first case and MongoDB-based in the second one) has been designed according to the above listed abstract concepts. The first level, residing in the Smart Home architecture (Figure 3), consists of a local DB, organized in different static and dynamic tables [29]. The static ones contain the lists, for the apartment of interest, of different users, devices, features and indicators to be computed. The dynamic tables are designed to collect all the raw quantities from sensors and the associated indicator values: each raw datum is univocally identified by the combination of three ID numbers (user, device, and feature). In addition, a computing bundle has been implemented to process the raw data for fixed time span (i.e., daily, weekly or monthly) and store the result of such process (e.g., average, trend, etc.) in a different dynamic table (the “Indicator values” table).

The second level consists of a Cloud DB. The Cloud exposes a web service and several REST APIs, (called H@H APIs after the name of the Health@Home project (Figure 3)) for letting, on the one hand, the data upload module periodically performs its upload tasks, and, on the other hand, authorized users access the data from remote. Authorized users include the monitored users themselves, and the authorized personnel conducting the Pilot study. Both the local and Cloud components are equipped with several layers of protection, including HTTPS connections, credential and token-based authentication, database encryption (at the Cloud level) or OS level encryption (at the local level), selective IP authorization, etc. In any case, data stored on both DBs are anonymous, since different users are just represented by alphanumeric users IDs. For the pilot case considered in this work, the local DB was used to temporarily store data extracted by the physiological devices. Conversely, data coming from the *Home Automation Kit* (i.e., collected through the *Home Automation App*, or service) was directly sent to the Cloud. The data acquired in the pilot case are also kept available, for a limited amount of time, on the Cloud database to allow their future processing and the development of dedicated services from the involved research teams.

### 2.4. User-Machine Interaction

**Figure 7:**
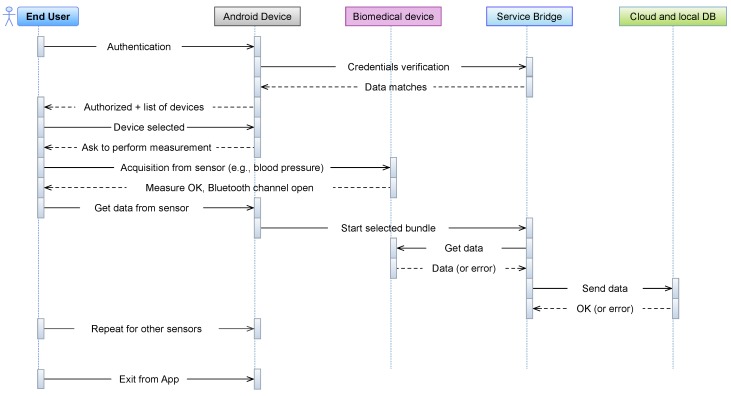
Overview of how the user interacts with the *Health App* and performs a measurement.

While the domotic quantities (e.g., PIR events and indoor air temperature) are collected automatically through the dedicated *Home Automation App*, the biomedical features require an interaction with the user. Considering the devices installed in each apartment, a description of the steps to be conducted for a single acquisition is reported in Figure 7.

**Figure 8:**
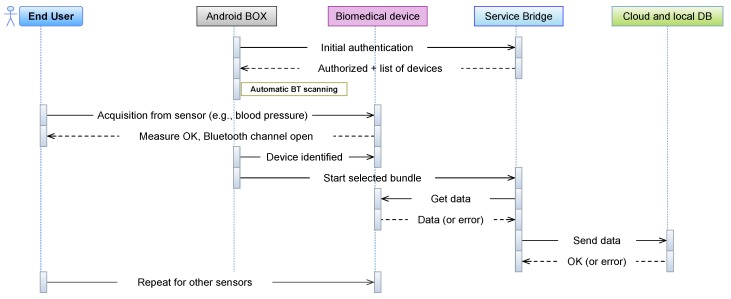
Modified user interaction with the Health@Home system.

First, the user has to interact with the Android device (i.e., *Health App*) and with each individual biomedical device. Once the App starts, the user proceeds with the authentication step, by means of the username/password credentials. The App interacts with the local web server in the *Service Bridge* and, if authenticated, the list of installed devices will be shown in the GUI of the App. Then, if the user chooses a sensor, a new window will popup, which asks the user to start the physical measurement procedure (e.g., the body weight). Notably, each user is presented with a personalized layout, including only those devices (sensors), among those installed in the house, which she/he is entitled to use. This is possible since the set of allowed devices is downloaded (from the *Service Bridge*) at each login. The information on which user can use which device is contained in a table of the local database of the *Service Bridge*. This approach has two advantages. First, it makes the interface more simple for elderly users: if one user is interested in blood pressure monitoring only, and another one in glycemia monitoring, each of them will see only the dedicated devices, making the interface simpler to use. Second, as a user decides to start a new kind of monitoring, e.g., acquiring a new device, or getting the permission to use a device already present in the house, but formerly used by another user only, there is no need to update the app. In fact, only the user permission table on the *Service Bridge* needs to be updated and, if the device is an entirely new one, the corresponding bundle has to be installed in the *Service Bridge*, but this is completely transparent to the client app running on the Android device, which will just get the updated information, from the *Service Bridge*, upon login. Each BLE device adopted, once the measure has been conducted, will open the Bluetooth communication channel, meaning that data are available to be collected. While the user selects the “get data” button from the *Health App*, the same App communicates the bundle to be executed to the *Service Bridge*, which starts collecting data from the sensors. If something goes wrong, an error message will be communicated to the bundle. Otherwise, data collected will be sent to the storage repositories (Cloud and local DB). The same procedure could be performed for each other biomedical device. Finally, the user can exit the App through a dedicated button. In summary, the user interaction with the *Health App* is reduced to these three steps: authentication, device acquisition trigger and exit from application. Even if these tasks seem simple to be conducted, the steps described must be performed sequentially, otherwise the acquisition procedure could generate errors, with potential data loss. In fact, some users felt comfortable in using the App and the procedure explained, while others found difficulties in interacting with the Android device. However, the totality of users was conscious of how to perform the biomedical measurements requested (i.e., body weight, pressure, temperature and blood saturation/HR). This suggested the authors to provide a simplification of the previous procedure, with the integration of an Android Box, as replacement of the smart device (i.e., without an App and a GUI). The adoption of the new device provided a modification in the user-machine interaction, which can be summarized in Figure 8.

## 3. Pilot Case

The H@H pilot case, presented in this paper, is Oderzo, a small town in Veneto Region, where the H@H consortium has identified, together with local entities, 13 older persons living in 8 apartments that have been steadily monitored. The identification of the pilot case has followed these 4 phases:

- *Phase #1*: definition of the cohort, started on July 2017 and finished after two months;
- *Phase #2*: installations, from December 2017 to January 2018;
- *Phase #3*: user instructions, from the end of January 2018 to February 2018; and
- *Phase #4*: tests, running phase.

**Figure 9:**
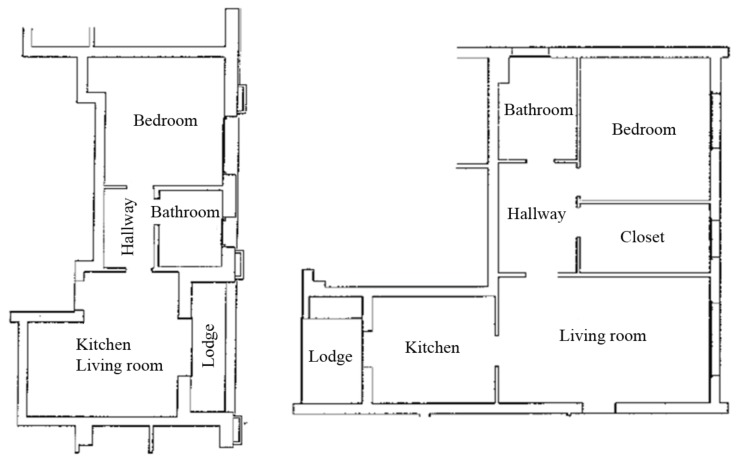
Schematic of plans for some apartments of the pilot case.

The decision to choose this cohort, *Phase #1*, is based on both user and apartment requirements. The requirements for the users are: age (65–85 years old), gender (both males and females), pathologies (not particular pathologies ), family group (user alone or couples), social status (normal) and psychological and physiological conditions (not psychological disease and ability to perform daily living activities). The apartment requirements are based on devices to be installed, electrical system to be replaced or installed as new and web connectivity for all apartments. The matching between the user and apartment requirements, defined the selected cohort for the considered pilot case in Veneto Region. *Phase #2* has been characterized from the collaborative work of the installers together with the project researchers to define the installation strategies for each apartment. Each apartment has a similar plan, so, the devices and sensors have been placed, taking into account the structural and implant characteristics of the apartments. Note that the installation of smart home equipment is comparable in each apartment (e.g., one PIR in the main bathroom, bedroom and living room, and thermostat in the living room). Two of the considered plans are shown in Figure 9.

*Phase #3* consisted on providing the instructions to the users for correctly making the measurements (i.e., biomedical equipment). Although the authors made a user manual with the line guide to acquire data and signals, a period of 2–3 days with the users was necessary to start with the measurement setup. The test phase, namely *Phase #4*, is a complex part of the considered pilot case, where the authors identified some problems in the user interaction with the technology. For this reason, the architecture of the smart home sensor network required some changes, described in previously Section 2.2.

### 3.1. Smart Data Processing

The proposed architecture, hardware and software were installed in the eight apartments in Oderzo, where some of these have been selected for data processing. Within the entire experimentation, the users’ behavior inside the house was constantly monitored (e.g., the light status monitoring, PIR activation, etc.), together with their health parameters, as described in previous sections. Then, the attention focused on how to process and combine such heterogeneous data, through machine learning techniques, to derive useful and high-level information. The authors identified two main aspects. The authors firstly describe the features acquired by the home automation equipment (domotic features) and by the biomedical device (biomedical features). These features were used as predictors of the machine learning model in the first data analysis (i.e., *Case I*). In particular, in *Case I*, the aim was to investigate the classification of different apartments, related to different human behavior using domotic, biomedical and domotic + biomedical features. Since in *Case I* both data modalities (i.e., domotic and biomedical features) used as predictors should have the same frequency (i.e., daily), the authors filled the missing biomedical data with zero-hold interpolation. The goal of *Case II* was to investigate within the single smart home the possibility to discriminate possible health alert events, taking as predictors only the domotic features. In particular, for the *Case II*, where the domotic features are taken as a predictor for the health status, the daily median HR and the average systolic pressure were clusterized. In this case, there was no need to fill the missing biomedical data, so no resampling strategy was performed.

#### 3.1.1. Case I: Classification of Users’ Behavior and Smart Home Profile

The first aspect is the classification of the different apartments, using classification approaches (i.e., supervised learning techniques), according to the acquired data. This kind of analysis is interesting for both assessing the correlation between the huge number of involved variables, and for identifying the subset of data (i.e., data reduction) that allows an accurate estimation of the different scenarios. This analysis has interested the application of different machine learning approaches (i.e., Decision Tree (DT) [33], Neighborhood Component Feature Selection (NCFS) [34], Support Vector Machine (SVM) [35]), considering only the home automation variables, the health ones, or both.

Since the sensor installation may slightly differ from one apartment to another (e.g., smart fridge installed in few houses), the analysis focused on the features monitored in common, i.e., for the domotic:

- *Indoor air temperature* (average, standard deviation, range, maximum and minimum values);
- *Light status*, as the total count of activation (i.e., living room, extractor hood, hallway, main bedroom, main bathroom, bathroom mirror); and
- *PIR*, as the total activation event occurred in a fixed time (i.e., living room, main bedroom, main bathroom).

Once the raw data of the home automation equipment were collected, they were manipulated to derive the features described above.

The same methodology was applied to the following acquired common biomedical quantities:

- *Heart rate* (average, standard deviation, range, minimum and maximum values);
- *Systolic pressure*;
- *Diastolic pressure*;
- *Mean Arterial Pressure*;
- *Oxygen saturation* (average, standard deviation, range, minimum and maximum values);
- *Body weight*; and
- *Body temperature*.

For these features, given the fact that the self-monitoring was generally performed 2–3 times a week (thus, fewer and asynchronous data with respect to the home automation ones), the missing values were filled, with a zero-hold approach. This approach allowed producing a synchronized matrix of features for the application of different classification techniques. However, since the filling of data may be excessive, in the case of hourly analysis, the daily processing approach has been preferred. The user’s behavior classification analysis, namely *Case I*, was conducted for five different homes, where sufficient home automation and biomedical data were collected.

#### 3.1.2. Case II: Classification of Health Condition from Home Automation Feature

The second analysis focused on the possibility to discriminate possible alert events for the user, from a smart processing of the home automation quantities. Thus, differently from the previous case, this analysis was intra-subject. The aim of such analysis was to evaluate if the user health status, according to preliminary information given by her/his self-monitoring of several biomedical features, can be deduced or predicted, with enough accuracy, by her/his behavior within the home. In this case, the target response is the normal condition versus possible alert situation, identified through data clustering of two biomedical features (i.e., unsupervised learning technique from daily median HR and average systolic pressure quantities). In particular, the *k*-means approach was used to clusterize the biomedical data [36], providing the corresponding annotation (i.e., normal versus alert condition). This analysis was conducted for three different cases:

- An apartment (namely, Home 1), where a couple lives (namely, User 1 and User 2) and biomedical data were collected for both. In this scenario, the focus is on assessing how the prediction analysis may react (i.e., it is not possible to discriminate the home automation features for the different users, while this can be performed for the biomedical quantities).
- An apartment (namely, Home 2), where a person lives alone and, thus both home automation and biomedical data were related only to her/him.
- An apartment (namely, Home 3), where a couple lives (namely, User 1 and User 2) and biomedical data were monitored only for one person. This case is interesting to see the accuracy of the prediction, in the case the home automation features may present a higher dispersion, given by the second person.

For this analysis, biomedical data (used for the identification of possible alerts) were not filled (i.e., raw quantities with no zero-hold or any additional processing). In addition to the approach discussed for the previous test, the data related to both light and PIR activations were also analyzed hourly (i.e., for each day the total count of events for each hour). This allowed obtaining more discriminating power for the classification of user’s health status.

## 4. Results

### 4.1. Classification of Users’ Behavior and Smart Home Profile

**Figure 10:**
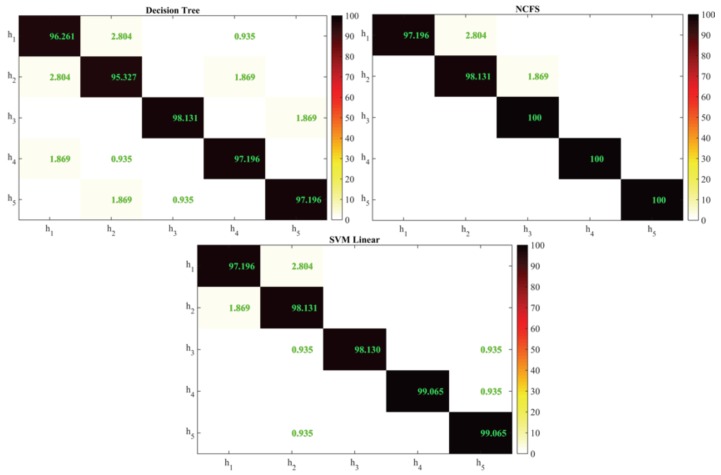
Confusion matrices for the different tested approaches. The classification has been obtained from the combination of health and home automation features, collected daily within the experimental trials in Oderzo.

In both experiments, the authors computed a 10-fold cross-validation procedure, averaging the results of all fold. Figure 10 shows the confusion matrices related to the first conducted analysis, i.e., the classification of the different user’s behavior and house profile using as predictors the home automation and biomedical collected features. The results come from a period of acquisition of three months.

Figure 10 highlights that the combination of several heterogeneous features allows discriminating the different user-house scenarios with high accuracy (i.e., >95% for all the cases). Going in details, it is possible to conduct the same test using only the home automation features, or only the biomedical ones. Table 2 summarizes the accuracies obtained when this analysis is applied.

**Figure 11:**
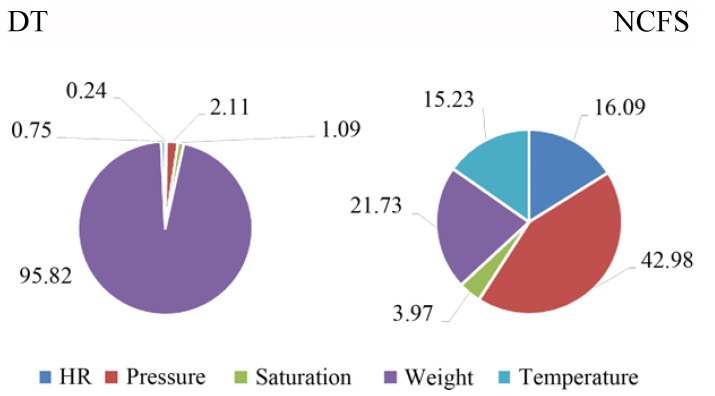
Percentage of importance of the health-related predictors for the classification of the users’ behavior and smart home profile.

Looking at the data, it can be noticed that classifying the users through biomedical features produces a higher accuracy with respect to their behavior (i.e., the home automation collected quantities). This is because the higher inter-subject discriminant power of the physiological features, with respect to the other case, while a higher variability occurs due to the impossibility to associate a behavior (e.g., bathroom light on) to a user respect to another in the same home. Another consideration is that there are some physiological quantities, e.g., the body weight, that alone may be able to discriminate the single users, if their values differs greatly. In this case, it is preferable to have a classification that considers more features than the mere body weight. Thus, a deeper investigation has been performed for the DT and NCFS applied techniques, to identify the most important predictors from the ones involved in the classification. Figure 11 shows the percentage of the importance index, calculated from the considered predictors.

**Figure 12:**
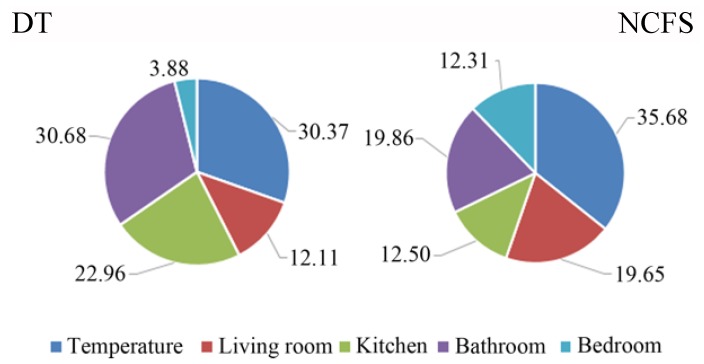
Percentage of importance of the home automation-related predictors for the classification of the users’ behavior and smart home profile.

As it can be noticed for the decision tree technique, it mostly considers the *Weight* feature for the classification, while, on the contrary, the NCFS method makes use of all the predictors. Thus, the second method should be preferred to the first one. Moreover, it is characterized by a higher accuracy with respect to the other tested methods. The same analysis has been conducted for the home automation features (see Figure 12), where it can be noticed that features related to the different scenarios (e.g., features related to the bathroom, as PIR triggers and lights on/off) are all important for a robust discrimination.

### 4.2. Classification of Health Condition from Home Automation Feature

The second analysis considered the possibility of evaluating possible health-related alerts, from a smart processing of the home automation quantities. First, within these months, no alerts were provided by the users; thus, the normal versus alert condition was derived by clustering the biomedical features, as said in the previous subsection. Then, the home automation features were used as the input for predicting the two group conditions (i.e., normal vs. alert) using a supervised classification approach (i.e., Decision Tree). The results of the proposed approach are summarized in terms of different classification metric in Table 3.

**Figure 13:**
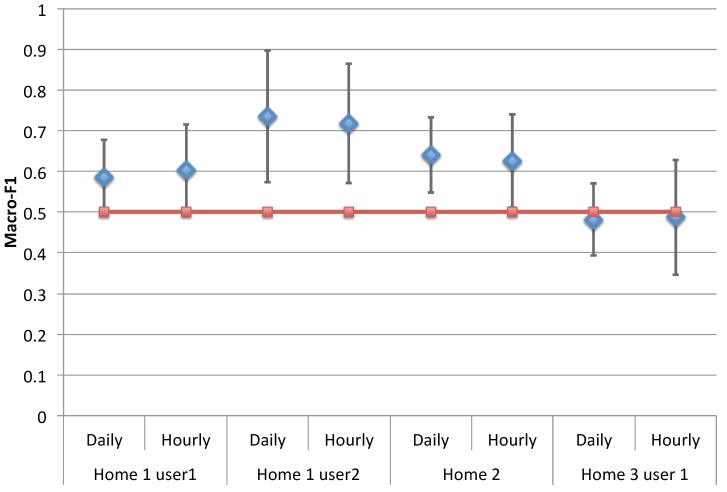
Macro-F1 scores.

A statistical comparison with respect to chance level was performed for the macro-F1 score computed for each fold of the cross-validation procedure. The macro-F1 is the F1 score averaged over each class. Classifier performance exceeds chance level (i.e., macro-F1 = 0.5) in Home 1, User 2 (mean=0.73, 95% CI = (0.573, 0.897), t9=3.29,p<0.01) and in Home 2 (mean=0.64, 95% CI = (0.548, 0.732), t9=3.44,p<0.01), while it does not overcome chance level in Home 1 User 1 (mean=0.58, 95% CI = (0.492, 0.678), t9=2.06, p=0.0694), and in Home 3 (mean=0.48, 95% CI = (0.393, 0.571), t9=−0.45,p=0.66). The obtained results suggest how the subject’s health condition is often encoded in the human behavior. For the single-resident analysis (i.e., Home 2), the results about the machine learning approach demonstrated how the situation of health alert is quite correlated to a change of human behavior captured by the domotic sensors. When there are multi inhabitants within the same apartment (i.e., Home 1 and Home 3), the human behavior is not always a good predictor of situation of health alert. This can be related to: (i) the difficulty to discern every single human behavior according to the domotic features; and (ii) the fact that the human behavior of one user may not be correlated to the situation of health alert of the other user. This is confirmed by the results represented in Figure 13 and Table 3. Although the best macro-F1 score is achieved (on average) by “Home 1, User 2”, which presents a higher variability especially with respect to the case of a single inhabitant (i.e., Home 2) (see Figure 13).

In fact, this is confirmed in the case of a single inhabitant (i.e., Home 2), where all the home automation features are strictly related to the same user. On the contrary, the machine learning model is not able to provide a clear distinction in the case of more persons living in the same apartment (i.e., in Home 1 and Home 3). This assumption is confirmed by the results of the performed analysis. However, the approach identified by the authors and discussed with these preliminary results is promising and will be better investigated and improved in future works. For example, the integration of an electronic logbook, where the users can provide information about their daily health status may be combined with the physiological features, in a semi-supervised manner, for improving the health condition’s prediction will be considered.

## 5. Conclusions

In this paper, the authors present the design and implementation of a smart communication architecture for user monitoring inside a domestic environment developed with an extensive partnership. The authors propose an innovative idea of an interoperable embedded intelligent system, where several simple and low-cost smart devices, both health sensors (*Physio Kit*) and home automation sensors (*Home Kit*), are used to monitor the general health status and the behaviors of elderly people. The chosen sensors are easy to use and non-invasive so that they can be easily accepted by an elderly person. One of the main innovative aspects of our proposed architecture with respect to the existing ones is the user interaction with the architecture in order to perform biomedical measurements. As for the hardware, in addition to the sensors, the project includes the use of a gateway as well as a smart device (i.e., a tablet) to display the App that interacts with the sensors. In addition, the App has been designed to be user-friendly, easy to use and acceptable to elderly users. In fact, one of the major problems encountered in AAL monitoring systems is precisely the non-acceptance or the inability to use the system itself by the user, especially when dealing with the elderly. The main goal that AAL systems must accomplish is to ensure the persons’ welfare, without, however, compromising the dignity of the person. Hence, extreme attention has been taken into account by the authors to this aspect of the system and, in this work, the authors propose an active approach to maintain the *Service Bridge* as transparent as possible, which means that user interactions are kept to the minimum required. An Android box has been installed in some apartments, which hosts the home automation service that communicates directly with the *Service Bridge* without the need of sending a command to start the biomedical parameters acquisition. In this way, the acquisition can be conducted by the user, without the need to interact with the *Health App* or, in any case, only for data visualization. In any case, a period of user training was necessary, which lasted approximately one month, since the combination of innovative technologies and elderly people requires time and efforts. The main target of the project was the development and the implementation of the software architecture taking into account in particular its modularity, scalability and extendibility, so the authors have decided to use an OSGi framework. The performance analysis of a system connected to many smart homes is another common problem of AAL monitoring systems, thus the use of a scalable architecture for storage, analysis, and sharing of many data is necessary. Moreover, the OSGi framework enables the integration of new sensors in a plug and play mode, only developing a new set of bundles without modifying the software architecture.

The feasibility and appropriateness of the proposed architecture and technologies in the creation of a low cost and flexible system has been successfully evaluated through an extensive experimentation. The entire hardware and software architecture was installed in eight apartments. Data from biomedical and home automation sensors were collected in the App and then sent to the Cloud where machine learning algorithms were used for the user behaviors identification and data analytics to finalize services for the elderly community. Experimentation has highlighted the stability of the novel adopted architecture and allowed obtaining some very promising preliminary results about two aspects: inter-subject classification, and intra-subject classification. In the first case, the classification of different house profiles using the combination of all features (both home automation and health) allows the discrimination of different house scenarios with high accuracy (mean±std=0.99±0.01) and macro-F1 score (mean±std=0.99±0.01). Moreover, an unsupervised approach was made to annotate data on which to carry out more in-depth studies in the future (e.g., PIR sensors will be used with the information of the timestamp to study user’s paths inside the house). In particular, in the second study case, the authors used home automation data to predict and distinguish normal and abnormal behavior. The machine learning algorithm learns the alert/normal condition from the clustering of health data and the results are very promising, especially when the person lives alone (i.e., macro-F1: mean=0.64, 95% CI = (0.548, 0.732), t9=3.44,p< 0.01). In the future, these machine learning algorithms could be used on the collected data to generate alarms when the activities carried out by the user and monitored by home automation sensors deviate from the typical normal behavior of the user. This could be a good solution for those elderly people who live alone and are not continuously assisted, as these alarms could be sent to a family member or a caregiver to alert her/him of a possible problem. In this way, the system could ensure an automatic management of alarms and allow detecting relevant information that cannot be inferred from data visualization.

## Figures and Tables

**Table 1:** Description of the devices part of the developed *Physio Kit*.

Device	Description	Accuracy	Resolution	Range
Zephyr Bioharness 3.0 (BH3)	Multi-parametric belt to monitor ECG and respiration signals, HR and BR values, acceleration, activity level and posture. Tests to evaluate the measurement accuracy for the ECG signal, heart and respiration rate monitoring were performed by the authors in a previous work.	HR: ± 1 bpm	HR: 1 bpm	HR: 25 ÷ 240 bpm
		BR: ± 1 bpm	BR: 0.1 bpm	BR: 3 ÷ 70 bpm
		Acceleration: n.a.	Acceleration: 0.012 g	Acceleration: −16 ÷ 16 g
		Posture: n.a.	Posture: 1${}^{{}^{\circ}}$	Posture: −180${}^{{}^{\circ}}$ ÷ 180${}^{{}^{\circ}}$
Taidoc TD3128B	Oscillometric blood pressure meter to monitor diastolic, mean and systolic blood pressure and HR.	Systolic: ±3 mmHg (±2%)	1 mmHg (Systolic, Diastolic)	Systolic: 60 ÷ 255 mmHg
		Diastolic: ±3 mmHg (±2%)	1 bpm (HR)	Diastolic: 30 ÷ 195 mmHg
		HR: ±4%		HR: 40 ÷ 199 bpm
Onyx Nonin 9560	Oximeter for the measurement of the oxygen saturation of blood and HR	Saturation: ±2%	Saturation: 1%	Saturation: 70 ÷ 100%
		HR: ±3 bpm	HR: 1 bpm	HR: 20 ÷ 250 bpm
Taidoc TD4277	Glucometer to analyze the glycemia values	±15 mg/dL (±15%)	1 mg/dL	100 ÷ 700 mg/dL
Taidoc TD1261C	Thermometer to measure the body temperature	±0.2 ${}^{{}^{\circ}}$C	0.1 ${}^{{}^{\circ}}$C	32 ÷ 43 ${}^{{}^{\circ}}$C
Taidoc TD2555B	Body weight scale to monitor the body mass weight	±0.3 kg (±0.5%)	0.1 kg	4 ÷ 250 kg

**Table 2:** Results of the users’ discrimination according to the quantities adopted in the classification.

	Accuracy (Average ± Deviation)		
**Classification Method**	**Health**	**Home Automation**	**Health + Home**
DT	0.97±0.03	0.86±0.03	0.97±0.03
NCFS	0.99±0.01	0.96±0.02	0.99±0.01
SVM	0.96±0.03	0.92±0.03	0.98±0.02

**Table 3:** Results of health condition classification.

Metric	Home 1, User 1		Home 1, User 2		Home 2		Home 3, User 1	
	Daily	Hourly	Daily	Hourly	Daily	Hourly	Daily	Hourly
True Positives (TP)	72.05	83.82	88.06	79.10	69.09	61.82	30.44	21.74
False Positives (FP)	27.94	16.18	11.94	20.90	30.91	38.18	69.57	78.26
False Negatives (FN)	50.00	59.38	36.36	30.30	40.00	35.56	28.57	23.38
True Negatives (TN)	50.00	40.63	63.64	69.70	60.00	64.44	71.43	76.62
Sensitivity (Recall)	59.04	58.54	70.77	72.30	63.33	63.49	51.58	48.19
Specificity	64.15	71.52	84.20	76.93	66.00	62.80	50.66	49.47
Positive predicted value (Precision)	72.06	83.82	88.06	79.10	69.09	61.82	30.44	21.74
Negative predicted value	50.00	40.63	63.64	69.70	60.00	64.44	71.43	76.62
False positive rate	35.85	28.48	15.80	23.07	34.00	37.21	49.34	50.53
False negative rate	40.96	41.46	29.23	27.70	36.67	36.52	48.42	51.82
Likelihood ratio positive	1.65	2.06	4.48	3.14	1.86	1.71	1.05	0.95
Likelihood ratio negative	0.64	0.58	0.35	0.36	0.56	0.58	0.96	1.05
Macro-F1	0.58	0.60	0.74	0.72	0.64	0.63	0.48	0.49

## Abbreviations

The following abbreviations are used in this manuscript:

API	Application Programming Interface
BLE	Bluetooth Low Energy
BP	Blood Pressure
BR	Breathing Rate
BW	Breathing Waveform
DB	DataBase
DT	Decision Tree
ECG	Electrocardiography
GUI	Graphical User Interface
HR	Heart Rate
HTTP	HyperText Transfer Protocol
HTTPS	HyperText Transfer Protocol Secure
ICT	Information and Communications Technology
IR	Infrared Radiation
NCFS	Neighborhood Component Feature Selection
OS	Operating System
OSGi	Open Service Gateway Initiative
PIR	Passive InfraRed
REST	REpresentational State Transfer
SOA	Service Oriented Architecture
SVM	Support Vector Machine
